# Utility of network integrity methods in therapeutic target identification

**DOI:** 10.3389/fgene.2014.00012

**Published:** 2014-02-03

**Authors:** Qian Peng, Nicholas J. Schork

**Affiliations:** ^1^Department of Molecular and Experimental Medicine, The Scripps Research InstituteLa Jolla, CA, USA; ^2^Scripps Genomic Medicine, The Scripps Translational Science InstituteLa Jolla, CA, USA

**Keywords:** network analysis, centrality, cancer, pathway, drug targets, personalized treatment, gene expression

## Abstract

Analysis of the biological gene networks involved in a disease may lead to the identification of therapeutic targets. Such analysis requires exploring network properties, in particular the importance of individual network nodes (i.e., genes). There are many measures that consider the importance of nodes in a network and some may shed light on the biological significance and potential optimality of a gene or set of genes as therapeutic targets. This has been shown to be the case in cancer therapy. A dilemma exists, however, in finding the best therapeutic targets based on network analysis since the optimal targets should be nodes that are highly influential in, but not toxic to, the functioning of the entire network. In addition, cancer therapeutics targeting a single gene often result in relapse since compensatory, feedback and redundancy loops in the network may offset the activity associated with the targeted gene. Thus, multiple genes reflecting parallel functional cascades in a network should be targeted simultaneously, but require the identification of such targets. We propose a methodology that exploits centrality statistics characterizing the importance of nodes within a gene network that is constructed from the gene expression patterns in that network. We consider centrality measures based on both graph theory and spectral graph theory. We also consider the origins of a network topology, and show how different available representations yield different node importance results. We apply our techniques to tumor gene expression data and suggest that the identification of optimal therapeutic targets involving particular genes, pathways and sub-networks based on an analysis of the nodes in that network is possible and can facilitate individualized cancer treatments. The proposed methods also have the potential to identify candidate cancer therapeutic targets that are not thought to be oncogenes but nonetheless play important roles in the functioning of a cancer-related network or pathway.

## 1. Introduction

Treating many forms of cancer effectively is notoriously difficult as most tumors have complex cellular dysfunctions replete with compensatory and redundancy mechanisms that contribute to tumor growth despite some aspect of the tumor being targeted for destruction by an anti-cancer therapeutic agent. Thus, while many cancer treatments seem effective when first administered, relapses often occur, particularly in later stages of tumor development. This general “robustness” of biological networks in tumor cells presents true challenges for cancer treatments and cures, especially if treatments administered only target a single gene. To reduce the likelihood of resistance and the risk of relapse, it may be important to target multiple pathways and oncogenes simultaneously, but the best way to do this has not been established (Hughes, 2007; Petrelli and Giordano, 2008; Dar et al., 2012).

While many tumors have certain pathologies and dysfunctional pathways in common, the specific mechanisms contributing to the growth of any one tumor are often distinctive and subtle. However, the identification of these mechanisms and the characterization of their contributions to individual tumor growth and treatment resistance can be greatly aided through the use of modern genomic assays and pathway analyses. Assays such as DNA sequencing, RNA sequencing, copy number variation assays, and proteomic profiling can reveal phenomena such as damaging mutations in oncogenes, resistance gene amplifications, and abnormal silencing of tumor suppressor genes. In conjunction with these assays, network and pathway analyses methods can reveal connections between different perturbations in tumors and may suggest interactions between genes that, if targeted simultaneously with different therapeutic compounds, could disrupt the network integrity of the tumor cells and lead to more effective interventions.

The best way to assess connections between multiple perturbations in tumors that could be targeted simultaneously is an open question. However, analyses of the principal properties, behavior and structures associated with biological networks within tumors may lead to the identification of more optimal therapeutic targets. Of the measures that one could consider in evaluating the properties of a tumor gene network, those focusing on network integrity are of particular interest. Network integrity analysis can lead to the identification of central gene nodes or gene *hubs* within the network that contribute to the maintenance and growth of a tumor in critical ways (Jeong et al., 2001; Ágoston et al., 2005; Perumal et al., 2009; Horvath, 2011; Li et al., 2011). For example, genes that are critical to the formation and growth of tumors have been observed to code for proteins that have increased levels of *connectedness* with other genes as well as greater *centrality* (i.e., occupying a more central place in the network rather than being on the periphery of the network) than genes that do not contribute to tumor growth and formation (Jonsson and Bates, 2006; Sun and Zhao, 2010; Xia et al., 2011). However, it has also been shown that most disease genes do not necessarily code for proteins that are hubs within a network, suggesting that some network characteristics may be better indicators of optimal therapeutic gene targets than others (Goh et al., 2007). In addition, most network analyses have been performed on comprehensive and generic interaction information rather than on networks or pathways specific to individual tumors, calling into question which type of network topology or representation an analysis should be pursued with. It is noteworthy, however, that network centralities have also been used to derive integrated gene signatures for breast cancer (Wang et al., 2011) and, in the context of signaling pathways, centrality-based analysis approaches have been used to identify enriched pathways from gene expression data (Gu et al., 2012), suggesting that different data types and approaches may provide complementary insights.

**Network Inference**

- Q: What types of biological networks have been inferred in the paper?
- A: We use gene expression data in conjunction with cancer-related signaling pathways to infer tumor–specific networks. The extracted tumor-specific networks help us further infer critical nodes (genes) and potential therapeutic targets for specific types of tumors or tumor cells.
- Q: How was the quality/utility of the inferred networks assessed?
- A: We compare and contrast the predictions with those derived using canonical pathways. We further compare the predictions on various normal tissues, tumor types and tumor cells. We also assess the results using multiple pathway/network databases.
- Q: How were these networks validated?
- A: Many of the targets predicted from the networks have supporting evidences in the literatures: they are either implicated as oncogenes or known targets of cancer treatments.

We assess the properties and characteristics of a cancer network topology based on gene expression data across a variety of tumors with subsequent analyses confined to specific types of tumors or tumor cells. We contrast the results of the use of different measures of network integrity on the ability to identify therapeutically meaningful gene targets in cancer networks. Our ultimate goal was to determine if it is possible to make compelling claims about the existence of gene targets that might be optimal for therapeutic intervention based on the network characteristics. We rank genes (i.e., nodes in the network) and edges based on their influences on network function and topology defined by various measures, and illustrate that centrality analysis on signaling pathways may provide additional insights to that based on protein-protein interaction (PPI) networks. One of the potential uses of network topology analyses like those we pursued is to identify targets that are not necessarily known to be directly cancer-related but may influence tumor growth nonetheless. Thus, in addition to common measures of network centrality which focus on cancer-related genes, we also investigate the utility of centralities based on spectral graph theory, including spectral gap centrality, that consider network function in a broader context and that have not been explored in the context of biological networks to date.

The remainder of the manuscript is organized as follows. Section 2 describes several centrality measures based on both graph theory and spectral graph theory, as well as the construction of network centralities based on gene expression data. Section 3 contrasts the critical nodes (i.e., genes) and edges defined and determined by different measures in cancer PPI subnetworks and pathways, pathways from different sources, and pathways conditioned on specific tissues and tumor cell lines. Section 4 summarizes the main observations and issues, and makes recommendations. We note that some of the terminology used in the literature and ways of referring to network components are often ambiguous. We use *network* and *pathway* interchangeably, although *network* often corresponds to the actual topology associated with a biological *pathway*. Also, when referring to *nodes* in a network (pathway) we are referring to individual *genes* and their place in the topology associated with a network (pathway).

## 2. Materials and methods

### 2.1. Critical nodes in a network

Network centralities are important structural attributes of a network. They can be exploited in analyses evaluating network robustness and reflect how much a network is connected and, importantly, how network functionality might be affected locally or globally if certain nodes or connections in the network are disrupted. There are many types of centrality measures (Freeman, 1978/1979; Koschützki and Schreiber, 2008; Horvath, 2011) and they are often used in different contexts. In biological network or pathway analysis, potential drug targets are expected to be highly influential nodes such that perturbing these nodes will have a major effect on network integrity and the flow of information through that network. These nodes might correspond to genes that affect many other genes in the network, or they could be associated with network fragility in the sense that if they are perturbed the network cannot function as a whole. Such highly influential nodes in a network or pathway might also be toxic to the entire network and lead to a complete inability of the network to function if perturbed. Such complete dysfunction might induce more harm than good if it is a network that normal, non-tumor cells require in order to function properly. In this light, it might be better to target nodes or genes that influence the most critical nodes in a network and not the actual critical nodes themselves. Among the various measures of network centrality that have been proposed in the literature, we primarily focused on the four measures described briefly below.

#### 2.1.1. Degree centrality

The simplest and the most common measure of node importance in the context of a specific network topology is degree centrality. Consider a network defined as a simple graph *G* = (*V*, *E*) with *n* = |*V*| nodes and |*E*| edges. The degree of node *v* ∈ *V* is the number of edges incident to *v*. Mathematically, the graph *G* can be represented as an adjacency matrix *A*(*G*), defined as

$$A_{ij}=\{\begin{array}{l} 1\text{ if }i,j\in V,\{i,j\}\in E,\\ 0\text{ otherwise,} \end{array}$$

where 1 ≤ *i*, *j* ≤ *n*. Note that in discussions of the adjacency matrix, we will often refer to node *v*_*i*_ as node *i* and use these two notations interchangeably. The degree centrality of node *i* is then defined as *c*_*d*_(*i*) = ∑_*j*_*a*_*ij*_ and reflects how well a node is connected as well as its likely direct influence on its neighbors.

#### 2.1.2. Betweenness centrality

The betweenness centrality is defined as the frequency with which a node is on the shortest path between two other nodes (Freeman, 1978/1979). It reflects the likely *control of communication* between other nodes by the node in question. There are definitional and operational differences between two types of betweenness centrality measures: *node betweenness* and *edge betweenness*. Betweenness for node *k* is defined as following,

$$c_{b}(k)=\sum_{i<j}\frac{g_{ikj}}{g_{ij}}$$

where *g*_*ij*_ denotes the number of shortest paths between nodes *i* and *j*, and *g*_*ikj*_ denotes the number of shortest paths between *i*, *j* through node *k*. Betweenness for edge *e* is similarly defined as,

$$e_{b}=\sum_{i<j}\frac{g_{iej}}{g_{ij}}$$

where *g*_*iej*_ denotes the number of shortest paths between nodes *i*, *j* through edge *e*. In contrast to the local effect of degree centrality, betweenness captures local connectivity as well as a node's global importance to the network. A node or edge of high betweenness essentially serves as a gatekeeper that could control the flow of information across the network.

#### 2.1.3. Eigenvector centrality

The eigenvector centrality is defined as the centrality of a node that is proportional to the sum of the centralities of the nodes it is connected to Bonacich (1972). The eigenvector centrality of node *i* is

$$c_{e}(i)=\frac{1}{\lambda }\sum_{j}a_{ij}c_{e}(j)$$

where λ is the largest eigenvalue of the adjacency matrix *A*. It reflects how well a node is connected to the well-connected nodes and how differences in node degrees propagate through a network. Both Google's PageRank measures and Katz centrality are variants of the eigenvector centrality.

#### 2.1.4. Spectral gap centrality

Another measure derived from spectral graph theory was proposed by Wehmuth and Ziviani (2011). As it is based on the spectral gap of sub-networks, we will refer to it as *spectral gap centrality*. The *diagonal degree* matrix of *G*, denoted *D*(*G*), is defined as

$$D_{ij}=\{\begin{array}{ll} d_{k} & \text{if }i=j=k,\\ 0 & \text{otherwise,} \end{array}$$

where *d*_*k*_ is the degree of node *k*. The normalized Laplacian matrix (Chung, 1997) of graph *G*, denoted *L*(*G*), is defined as

$$L_{ij}=\{\begin{array}{ll} 1 & i=j,\\ -\frac{1}{\sqrt{d_{i}d_{j}}} & \{i,j\}\in E,\\ 0 & \text{otherwise.} \end{array}$$

All eigenvalues of *L*(*G*) are between 0 and 2, i.e., 0 = λ_1_(*L*) ≤ λ_2_(*L*) ≤ … ≤ λ_*n*_(*L*) ≤ 2. If *G* is a single connected component, λ_2_(*L*) (referred to as the *spectral gap*) is the smallest non-zero eigenvalue and is less than 1 if the graph is not complete. λ_2_ approaches 0 as the graph becomes less connected. The critical nodes are nodes with high spectral gap centrality. The spectral gap centrality of node *i* is defined as

$$c_{s}^{h}(i)=\{\begin{array}{ll} \frac{\lambda_{2}^{i}}{log_{2}(d_{i})} & d_{i}>1,\\ \infty & d_{i}=1. \end{array}$$

where λ^*i*^_2_ is the spectral gap of the *h*-neighborhood of node *i*, i.e., the subgraph induced by all nodes within *h* edges from node *i*, and *d*_*i*_ is the degree of node *i*. The lower the value *c*^*h*^_*s*_(*i*), the more critical the node *i* is to the network. The spectral gap centrality thus reflects the neighborhood connectivity, and captures both degree and betweenness to some extent depending on the value of *h*.

The four centrality measures are chosen primarily for their representative characteristics of networks, their direct relevance to potential biological functions that we are interested in, and the intuitive interpretation of the results. Among other measures that might be of interest, closeness (Sabidussi, 1966) and radiality (Valente and Foreman, 1998) centralities reflect how quickly a node can reach another, which represents a different type of functionality. Closeness centrality requires network to be strongly connected which is often not the case for pathways. PageRank (Page et al., 1999) and Katz status index (Katz, 1953) are variants of eigenvector centrality. Another class of centralities are motif-based (Koschützki and Schreiber, 2008), which represent functional substructures, thus are more likely employed in specific contexts.

### 2.2. The origins and relevance of network and pathway topologies

The question of which centrality measure yields a better prediction for therapeutic targets is only one of many important questions associated with biological network analyses. A more fundamental question is which biological network to interrogate. Network analysis can be applied to protein-protein interaction (PPI) networks, often derived empirically through experimentation, or biological pathways that have been described over the years. The choice of a particular pathway is also complicated, since there are multiple versions and subcomponents of pathways to choose from. One option is to derive a protein-protein interaction subnetwork from the genes of relevance to a particular, e.g., phenotype that are grounded in a pathway. An alternative is to analyze the pathway topology directly without considering the elements associated with a protein-protein interaction subnetwork. Different choices of a network or pathway representation—even if chosen to address the same overarching questions—will undoubtedly yield different results due to intrinsic differences between PPI subnetwork definitions and pathways. In addition, the same pathway defined from different database sources, or compiled based on different readings or reviews of the literature, may also yield different results due to topological differences between the network representations. Further compounding these issues is the fact that all genes in a pathway are not equally expressed in all tissues. Thus, networks constructed from one set of resources or experiments may not represent the true network topologies associated with different tissues. For the identification of critical nodes and genes to be relevant to a particular biological setting, tissue-specific network configurations might need to be considered. Obviously, if a gene is not expressed in a particular tissue of interest, for example, the node in another tissue-derived gene expression-based network corresponding to that gene and its associated edges must perforce be deleted from the network, thus altering the network topology.

To evaluate the effects of different network representations and different network centrality measures on the identification of critical nodes in that network, we analyzed MAPK and EGFR signaling pathways and configurations obtained from different sources. We treated these signaling pathway representations as true networks. We obtained pathway information from the KEGG (Kanehisa and Goto, 2000) and WikiPathways (Pico et al., 2008) databases. We obtained a human PPI network from the STRING (Mering et al., 2003) database. In order to have the pathway representations comparable to PPI network representations, we treated them as undirected graphs. Note that the PPI subnetwork from a pathway is a subgraph of the entire PPI network limited to the nodes corresponding to the intersection between genes implicated in the pathway and those present in the PPI network.

To compare and contrast tissue-specific pathways (based on the genes expressed in that tissue) and more generic, non-expression-based pathways, we analyzed cancer-related pathways based on expression patterns obtained from the NCI60 tumor cell lines (Scherf et al., 2000). To determine expression patterns in the NCI60 cell lines, we applied the Gene expression barcode algorithm (McCall et al., 2011) to the Affymetrix gene expression data of each cell line, which yielded an expression state (i.e., expressed/unexpressed) for each gene in each cell line. In addition, we analyzed pathways conditioned on a set of gene expression states and levels obtained from normal tissues. RNA-Seq data for eleven human tissues were obtained from RNA-Seq Atlas (Krupp et al., 2012). A threshold on gene expression value RPKM (reads per kilobase of transcript per million mapped reads (Mortazavi et al., 2008)) was used to filter genes such that genes with expression levels having an RPKM < 0.5 were considered unexpressed. Tissue or cell-specific pathway information was obtained by removing genes (i.e., nodes in the network) corresponding to unexpressed genes from the default pathway.

For each pathway (represented as a network) and each network centrality measure, the nodes (i.e., genes) within them were ranked in two ways: (i) by their centrality values; (ii) by the order that they were removed based on an iterative procedure to identify their importance in the network. This iterative procedure worked by removing top-ranked nodes based on centrality value (along with edges incident to the node), reassessing the nodes in the network and repeating this process until all nodes were assessed.

## 3. Results

### 3.1. The EGFR and MAPK pathways in cancer

We ultimately analyzed two different pathways known to have pronounced roles in oncogenesis: The epidermal growth factor receptor (EGFR) pathway and the mitogen-activated protein kinase (MAPK) pathway. Both EGFR and MAPK signaling pathways are well-studied and comprehensively curated, making them ideal for our comparison of various methods for assessing node importance and therapeutic target potential. We briefly describe each below.

EGFR, also called ErbB1, is a member of the ErbB family of receptor tyrosine kinases. The EGFR pathway is one of the most important pathways regulating cell growth, differentiation and survival (Holbro and Hynes, 2004). Abnormally high levels of the EGFR protein are frequently found on the surface of many types of cancer cells, facilitating the excessive cell division that is the hallmark of cancer. The defective regulation of the EGFR signal transduction pathway is also known to be associated with oncogenesis. EFGR and its signaling components therefore offer promising therapeutic targets for various cancers (Citri and Yarden, 2006; Scaltriti and Baselga, 2006).

The MAPK superfamily includes well-conserved kinase genes known to be involved in various cellular functions including cell growth, proliferation, differentiation, migration and apoptosis. They are regulated by four distinct groups of genes in mammals: ERK1/2, JNK, p38 and ERK5. While ERK1/2 and ERK5 pathways are relatively insulated, JNK and p38 kineses share many of their activators, thus the two cascades are more entangled (Chen et al., 2001; Yang et al., 2003). It has been well-established that aberrations in MAPK signaling play critical roles in cancer development and progression (Dhillon et al., 2007).

### 3.2. PPI sub-network versus signaling pathway analyses

The EGFR network we derived from WikiPathways [EGFR signaling pathways (Pico et al., 2008; Kandasamy et al., 2010)] has 235 nodes and 249 edges. The average node degree is 1.06, and the graph density (i.e., the fraction of possible edges) is 0.01. The PPI subnetwork induced by EGFR pathway has 119 nodes and 4638 edges. Its average node degree is 39, and the graph density is 0.66. We applied the different centrality measures discussed above to each network and ranked the nodes (genes) on the basis of these measures. Tables 1, 2 list the top-ranked genes (ranked between 1 and 10 for at least one measure) obtained from the PPI subnetwork and the EGFR pathway, respectively. The left five columns reflect degree centrality, node betweenness, eigenvector centrality, spectral gap centrality with *h* = 2 and spectral gap centrality with *h* = 3; the right five columns provide corresponding measures with top-ranked nodes removed and the remaining subgraphs re-evaluated iteratively. The upper triangular matrix at the bottom half of the table gives Spearman's rank correlation coefficients assessing the relationship between the results of each pair of metrics. This is computed using the actual rankings of all genes listed in the table, including those ranked beyond ten.

**Table 1:** **Top ranking genes in EGFR PPI subnetwork by various centrality measures**.

**Gene**	***c*_*d*_**	***c*_*b*_**	***c*_*e*_**	***c*^2^_*s*_**	***c*^3^_*s*_**	***c*^*r*^_*d*_**	***c*^*r*^_*b*_**	***c*^*r*^_*e*_**	***c*^2*r*^_*s*_**	***c*^3*r*^_*s*_**
AKT1	1	1	1	1	1	1	1	1	1	1
EGF	2	3	2	2	2	2	3	2	2	2
EGFR	3	2	3	3	3	3	2	3	3	3
GRB2	4	5	4	4	4	4	5	5	5	4
MAPK1	5	4	5	5	5	5	4	4	4	5
RAC1	6	7	6	6	6	6	8	6	6	6
CDC42	7	6	7	7	7	7	6	8	7	7
MAPK3	8	10	8	8	8	8	10	7	8	8
STAT3	9		9	9	9	9		9	9	9
ERBB2	10	8		10	10	10	7		10	10
FOS		9					9			
PTEN			10					10		
*c*_*d*_		0.92	0.97	1	1	1	0.9	0.96	0.99	1
*c*_*b*_			0.87	0.92	0.92	0.92	0.99	0.85	0.93	0.92
*c*_*e*_				0.97	0.97	0.97	0.83	0.99	0.97	0.97
*c*^2^_*s*_					1	1	0.9	0.96	0.99	1
*c*^3^_*s*_						1	0.9	0.96	0.99	1
*c*^*r*^_*d*_							0.9	0.96	0.99	1
*c*^*r*^_*b*_								0.8	0.9	0.9
*c*^*r*^_*e*_									0.97	0.96
*c*^*r*2^_*s*_										0.99

*c_d_, degree centrality; c_b_, node betweenness; c_e_, eigenvector centrality; c^2^_s_, c^3^_s_, spectral gap centrality h = 2, 3. c^r^_{d,b,e,s}_,c^{2,3}r^_s_, node ranking are obtained by consecutively removing the top ranked nodes. The bottom matrix is Spearman's rank correlation coefficients*.

**Table 2:** **Top ranking genes in EGFR pathway by various centrality measures**.

**Gene**	***c*_*d*_**	***c*_*b*_**	***c*_*e*_**	***c*^2^_*s*_**	***c*^3^_*s*_**	***c*^*r*^_*d*_**	***c*^*r*^_*b*_**	***c*^*r*^_*e*_**	***c*^2*r*^_*s*_**	***c*^3*r*^_*s*_**
SRC	1			2	5	1	2	3	2	5
STAT3	2		1	3	2	2		1		2
EGFR	4		3	1	6	7	7		1	10
HRAS	6	3			8	5	9	4	6
MAPK1	7	9		4	7	6	6		3	4
MAPK3	8				9	9		2
GRB2	9	1		7	1	8	1	5	5	1
MAPK7	10					10				8
SOS1		2		6	3
RAF1		4		5			3		4
REPS2		5
ASAP1		6					4
MAP2K1		7			4					3
MAP2K2		10						6
STAT1			2
JAK2			4
JAK1			5
PIAS3			6
COX2			7
GRIM19			8
PLCG1				9					8	9
GAB1							8
PLD1							10
CBLC								9
MAPK8								10
SH3KBP1									7	7
JUN									9
JUND									10
*c*_*d*_		0.34	−0.27	0.76	0.68	0.87	0.53	0.76	0.66	0.69
*c*_*b*_			−0.35	0.65	0.78	0.3	0.44	0.26	0.4	0.39
*c*_*e*_				−0.29	−0.25	−0.19	−0.31	−0.24	−0.16	−0.21
*c*^2^_*s*_					0.81	0.53	0.5	0.49	0.71	0.51
*c*^3^_*s*_						0.65	0.44	0.49	0.48	0.7
*c*^*r*^_*d*_							0.65	0.8	0.48	0.79
*c*^*r*^_*b*_								0.6	0.54	0.45
*c*^*r*^_*e*_									0.35	0.5
*c*^*r*2^_*s*_										0.51

Spearman's rank correlation coefficients are computed on genes in the table with their actual rankings (ranking > 10 not shown). Nodes that do not directly correspond to genes are omitted.

As shown by Spearman's ρ in Table 1, the rankings from different metrics are highly correlated among genes in the PPI subnetwork. This can be explained by the properties of the network. The PPI network, like many biological networks (Lima-Mendez and van Helden, 2009), has the following properties: (i) High-degree nodes tend to be connected with other high-degree nodes; (ii) The network diameter (i.e., the length of the longest of the shortest paths between any two nodes) is usually small. A subnetwork shares these properties if it is induced on nodes of high degrees. Genes corresponding to high-degree nodes in a PPI network usually have systemwide effects and are involved in multiple pathways including cancer-related pathways (Han et al., 2004; Barabási et al., 2011). Spectral gap centralities in such a subnetwork are largely dominated by node degrees, and eigenvector and betweenness centralities also track the degrees for the high-degree nodes. Consequently, different centrality metrics on a pathway-induced PPI subnetwork are unlikely to yield significant insights beyond what is already coded in node degrees. As noted, although high-degree nodes in a PPI network may serve as effective drug targets, they are also likely to be toxic if perturbed in severe ways, due to their system-wide influence, i.e., their likely being involved in many cellular functions as they influence many pathways simultaneously. In this light, Wang et al. (2013) showed that the number of side effects of a drug is positively correlated with the degree and betweenness centralities of that drug's targets in the protein-protein interaction network. This observation was found to be the case for both cancer and non-cancer drugs.

In contrast to an analysis of the PPI network, the different centrality measures produced different node rankings when applied to the pathway information, as described in Table 2. Thus, while some nodes ranked high in multiple metrics indicating their overall importance, there are groups of nodes that rank high based on one or another measure, especially with respect to the betweenness and eigenvector centrality measures. High ranking nodes based on the betweenness and eigenvector centrality measures appear to be exclusive to each other in the pathways we have analyzed. The spectral gap centrality measure tends to capture a few nodes ranked high by each of the other three metrics. Similar results were observed when we analyzed the MAPK pathways, as described in the next section.

We note that genes (nodes) ranked high exclusively by the eigenvector centrality measure (i.e., STAT1, JAK2, JAK1, PIAS3, COX2, GRIM19) are all neighbors (directly downstream or upstream in the pathway) of STAT3, which plays a leading role in cancer inflammation and immunity, and is a validated target for cancer therapy (Yu et al., 2009). JAK-STAT signaling is a well understood cascade as its aberrant activation has been implicated in various types of leukemias, as well as solid tumors (Ferrajoli et al., 2006; Sansone and Bromberg, 2012). In addition, it has been established that STAT1 overexpression is associated with anticancer drug resistance (Khodarev et al., 2012). Interestingly, the FDA-approved drug ruxolitinib is a JAK1 and JAK2 inhibitor, and more JAK inhibitors are in development (Verstovsek et al., 2012). Also, PIAS3 overexpression has been shown to inhibit cell growth and increase drug sensitivity in lung cancer (Ogata et al., 2006), and several studies have indicated that COX2 inhibitors (NSAIDs and celecoxib) have protective effects against colorectal cancers and breast cancers (Gupta and DuBois, 2001; Arun and Goss, 2004; Brown and DuBois, 2005). Finally, Okamoto et al. (2010) demonstrated that overexpression of GRIM19 in cancer cells suppresses STAT3-mediated cancer growth.

As emphasized, the analysis of singular nodes that may be logical drug targets in a network is tremendously important in cancer therapeutic development. However, targeting multiple signaling pathways simultaneously is an essential strategy in managing cancer and reducing the possibility of an individual tumor developing drug resistance. It is therefore important to identify critical genes in multiple cascades within a network. By removing top-ranked nodes that appear to be the most critical for drug response and then re-evaluating the remaining subnetworks, additional critical nodes that may act as redundancy and compensatory mechanisms and contribute to drug resistance can be identified. Interestingly, when the betweenness and spectral gap centralities are applied to a network in such fashion, the first few critical nodes often reside on different paths (cascades) in that network. This phenomenon is not as pronounced for the node degree and eigenvector centrality measures, as their values are affected primarily by a node's nearest neighbours in the network and the properties that these neighboring nodes have. For example, consider *c*^*r*^_*s*_ as applied to the EGFR pathway (Table 2): for *h* = 2, the top three nodes are EGFR, SRC, and MAPK1 (ERK), belonging to two paths; for *h* = 3, the top nodes are GRB2, STAT3 and MAP2K1 (MEK), also on two cascades, the classical MAPK and Jak-STAT cascades. We observed similar effects in the analysis of the MAPK pathway as detailed in the next section. In this light, nodes ranked high by *c*^*r*^_*s*_ alone, such as SH3KBP1, PLCG1, JUN, JUND, might also serve as potential therapeutic targets. SH3KBP1 has been implicated in cell death and shown to mediate down regulation of EGFR (Soubeyran et al., 2002; Feng et al., 2011), and JUND has been shown to reduce tumor angiogenesis (Gerald et al., 2004). We consider the betweenness centrality measure in a separate section.

### 3.3. Different representations of the same network

There are often multiple sources for the same biological network or pathway. Variations in the topology of a network associated with different representations of that network can be attributed to, among other things: what genes or proteins (nodes) are included in the network; what types of interactions are included (e.g., gene-protein, protein-protein, interactions derived from correlations in expressions values of genes) and how they are represented as edges in the network; and how protein complexes are represented. The MAPK pathway can be used to illustrate this. The MAPK pathway from KEGG (Kanehisa and Goto, 2000) has 129 unique nodes and 161 edges (average node degree = 1.25; graph density = 0.02), while the same pathway from WikiPathways (Pico et al., 2008) is made of 186 nodes and 168 edges (average node degree = 0.90; graph density = 0.01). Note that a pathway is not always represented as one connected component. A main difference between the KEGG and WikiPathways representations is that protein-gene complexes are shown as single nodes in the former, while various components of the complex appear individually in the latter, and additional nodes, referred to as compound nodes subsequently, are used to group the complex together. Although the different representations of the MAPK pathway have biological appeal, since they exploit and incorporate different data types and ways of integrating them, the resulting topologies are quite different and obviously affect the ability to identify critical nodes in that pathway. For instance, nodes connecting a complex (i.e., compound nodes) in WikiPathways often have high degrees. Consequently, the significance of the individual nodes in the complex, as well as other nodes, will be affected in the identification of critical nodes. Pathways involving different data sources may be represented as compound graphs for perhaps a clearer layout and to facilitate more modularized modeling (Dogrusoz et al., 2005). However, it is unclear how best to treat differences between pathway representations in a network analysis, especially with respect to what makes the most biological sense, as well as how to interpret the different results. We considered analyses involving both the KEGG and WikiPathways representations to highlight differences that may result from their use.

Tables 3, 4 list critical nodes identified from the MAPK pathway as derived from the KEGG and WikiPathways representations. While some nodes ranked high in one pathway but not the other, most top-ranked nodes are shared. Their rankings, however, are rather different. Of the compound nodes in the MAPK pathway from WikiPathways, CASP^*^, PPP3^*^ and PRKC^*^ rank high essentially because they are each connected to multiple individual genes (7, 5, 5 genes, respectively) of a complex, thus having relatively high degrees. While compound nodes highly affect the degree centrality ranking, other measures, especially the spectral gap centrality measures, are less affected (unless average node-degree is high, as shown in the analysis of the PPI subnetworks), making them more informative and reliable. In addition, the spectral gap centrality measure, when applied with a higher *h*, captures nodes with more global rather than local importance. For instance, the top three nodes by *c*^*r*^_*s*_(*h* = 3) in the KEGG MAPK pathway representation are RAF1, ASK1 and MEKK1, which are on the ERK1/2, p38, and JNK cascades respectively. Similarly the top three nodes in WikiPathways MAPK pathway representation are ERK, MEKK1 and MKK7, which are on the ERK1/2 and JNK cascades. As shown in the previous section, nodes captured by eigenvector centrality are especially interesting, particularly if they are not captured by other measures, since they are often connected to otherwise critical nodes, thus suggesting that these nodes have the potential of being a direct influence on the behavior of the network. For instance, the MKP (from the DUSPs gene family) and PTP genes are ranked high by *c*_*e*_ alone and ranked 2 and 3 based on an analysis of the KEGG MAPK pathway representation, and as the top two *c*_*e*_ nodes in the WikiPathways representation of the MAPK pathway as well. These genes are known to be inhibitors of ERK, JNK and p38, thus covering three out of four potential cascades or crucial subcomponents of the MAPK pathway. Indeed, PTP genes have emerged as drug targets for cancer (Jiang and Zhang, 2008), and MKP-DUSP genes have been found to be involved in cancer progression and resistance, and have thus also become potential drug targets (Bermudez et al., 2010).

**Table 3:** **Top ranking genes in MAPK pathway (KEGG) by various centrality measures**.

**Gene**	***c*_*d*_**	***c*_*b*_**	***c*_*e*_**	***c*^2^_*s*_**	***c*^3^_*s*_**	***c*^*r*^_*d*_**	***c*^*r*^_*b*_**	***c*^*r*^_*e*_**	***c*^2*r*^_*s*_**	***c*^3*r*^_*s*_**
MEKK1	1	2	7	4	4	1	4	2	9	3
JNK	2	6	1	3	9	2	2	1	4	4
ASK1	3	7			7	3	3	7		2
Ras	4	4			3	4	8	4		5
ERK	5	5	6	10	5	5	6	6	7	7
Elk1	6		4			10		8
p38	7	10	5	7	8	6	5	3		6
GRB2	8	9			6	7		5		10
MAPKAPK	9			8					6
MKK7	10		10	9			10
MEK2		1		1	2		1		1
Raf1		3			1					1
SOS		8							10
MKP			2
PTP			3
MKK4			8	2					2
Sap1a			9
MKK3				5	10				5
cJUN				6
TNFR							7			9
IL1R								9
TRAF2									3
TAK1									8

**Table 4:** **Top ranking genes in MAPK pathway (WikiPathways) by various centrality measures**.

**Gene**	***c*_*d*_**	***c*_*b*_**	***c*_*e*_**	***c*^2^_*s*_**	***c*^3^_*s*_**	***c*^*r*^_*d*_**	***c*^*r*^_*b*_**	***c*^*r*^_*e*_**	***c*^2*r*^_*s*_**	***c*^3*r*^_*s*_**
CASP^*^	1	7		2	8	1		1
DUSP^*^(MKP)	2		2		9	2		4
TGFBR1/2	3			10		3	10	7	5
IL1R1/2	4	1		1	4		1	5	1	5
PPP3^*^	5					4	9	8		7
K/N/MRAS	6		6			5	3	6	9
PRKC^*^(PKC)	7					6
GRB2	8					7	7	10	7
MAP3K1(MEKK1)	9	2		4	2	8	4	3		2
MAPK8-10(JNK)	10		4	7		9
TRAF2		3		6	3
MAP3K7(MKK7)		4			6					3
MAP3K7IP1		5
RAC1/2,CDC42		8		5	10				3
PTPN5/7,PTPRR(PTP)		9	1	3	5		2	2	2	6
MAP2K4(MKK4)		10							10
MAPK1/3/4/6(ERK)			3		1		5			1
PTPN5			5	8
MAPK12-14(p38)			8			10	8
MAPK13			9
PTPN7			10
NRAS					7					4
IKBKB/G,MAP3K14								9		10
MAPK1									8
KRAS										8
MAPK10										9

Nodes that do not correspond to genes directly are omitted.

The PPI subnetworks associated with the MAPK pathway, based on both KEGG and WikiPathways representations, share similar properties with those from EGFR pathways: (i) the average node degrees are high; (ii) the graph densities are roughly 2/3; (iii) the node degrees dominate the critical node rankings of various metrics. As a result, the top-ranked nodes are the usual suspects, such as AKT1, P53, and RAF1. And for brevity's sake, we do not provide detailed descriptions of the results of this analysis.

### 3.4. The important role of betweenness centrality in network analyses

Since attacking multiple networks and pathways therapeutically in cancer is appropriate and necessary, it is imperative to find the critical signaling and major parallel cascade subnetworks. Betweenness centralities have the potential to reveal gatekeeping nodes or edges that control the flow of signal transduction along the cascades. In addition to node betweenness, edge betweenness may reveal targets that confer distinct functional advantages. It is noteworthy that although many genes/nodes in a network can be linked to multiple functions, it may be the case that only one of such links is disease-related (Zhong et al., 2009). Thus, blocking or perturbing a node with multiple functions may have unanticipated effects. The edge betweenness measure may offer more information than node importance in this regard. In addition, it could identify edges connecting major cascades involved in multiple functions. Since it is known that some cancer-related genes and proteins are difficult to target with small molecules, for example the p53 gene, drugs targeting an edge/interaction for which such genes are connected may offer ways of indirectly targeting and influencing those genes (Arkin and Wells, 2004).

Our analyses involving betweenness centrality with consecutive removal of top-ranked nodes or edges is more revealing. For instance, in Table 2, the following nodes with high betweenness, GRB2, SOS1, HRAS, RAF1, MAP2K1, MAP2K2, and MAPK1 are all on the same path. If the top-ranked node is removed and betweenness is re-evaluated on the remaining network, we immediately recognize the critical importance of nodes GRB2 and SRC, which are involved in multiple signaling paths in the network. Similarly, for analyses involving the edge betweenness centrality for the EGFR pathway, while four out of the top five edges by *e*_*b*_ are on the same path, the top five edges by *e*^*r*^_*b*_ are on four distinct paths (Table 5).

**Table 5:** **High betweenness edges in EGFR pathway**.

**Rank**	***e*_*b*_ (Edge)**	**Path**	***e*^*r*^_*b*_ (Edge)**	**Path**
1	GRB2–SOS1	Classical MAPK	GRB2–SOS1	Classical MAPK
2	SOS1–HRAS	Classical MAPK	SRC–PLCG1	Calcium
3	GRB2–REPS2		SRC–GAB2	SRC/GAB2/PI3K/AKT (Phagocytosis)
4	HRAS–RAF1	Classical MAPK	RAF1–MAP2K1	Classical MAPK
5	RAF1–MAP2K1	Classical MAPK	ASAP1–ARF6	PAG3/ARF6 (Phagocytosis)

This phenomenon of nodes and edges gaining or losing importance depending on the measure used is even more pronounced in the analysis of the MAPK pathways (Tables 3, 6). The MAPK pathways include four cascades: classical MAPK pathway (also known as ERK1/2 pathway), JNK and p38 MAPK pathway, and ERK5 pathway. The top three nodes by *c*^*r*^_*b*_, MEK2, JNK, ASK1, are on three of these cascades (Table 3). Table 6 suggests that while four of the top five edges by *e*_*b*_ are on the same ERK1/2 cascade, the top three edges by *e*^*r*^_*b*_ are each on one cascade: Raf1−MEK2 on ERK1/2, ASK1−MKK2 on p38, MKK−JNK on JNK, while the fourth edge MEKK1−MEK2 connects the JNK and ERK1/2 paths (see Xu et al., 1995 for details on this link). Thus, these nodes and edges do indeed essentially capture the main paths in the MAPK network. Note that the ERK5 cascade is presented as a separate component and the subgraph is a linear graph. Consequently, none of its nodes or edges ranked high in this particular analysis.

**Table 6:** **High betweenness edges in MAPK pathway (KEGG)**.

**Rank**	***e*_*b*_ (Edge)**	**Path**	***e*^*r*^_*b*_ (Edge)**	**Path**
1	Raf1–MEK2	Classical MAPK	Raf1–MEK2	Classical MAPK
2	Ras–Raf1	Classical MAPK	ASK1–MKK3	p38 MAPK
3	MEKK1–MEK2		MKK4–JNK	JNK MAPK
4	MEK2–ERK	Classical MAPK	MEKK1–MEK2
5	SOS–Ras	Classical MAPK	MKK4–MKK7	JNK MAPK

### 3.5. Pathway analyses conditioned on expressed genes in tissues and tumor cells

Not all genes are expressed in all tissues and cells. In tumor cells, certain genes are amplified, others silenced, often abnormally so. Not only do tumor cells differ from normal cells in this regard, but they also differ from each other. As such, the same pathway manifests differently in different cell types: if a gene is unexpressed, the encoded protein should be considered non-functional, and should be factually deleted from the pathway for an analysis. While analyzing the default pathway topology yields invaluable insights, tissue or cell-specific pathway topology needs be considered for network analysis to be more relevant. The best way to construct appropriate networks for cell or tissue-specific analyses is an open question, but might be achieved best by constructing them de novo from relevant experimental data (Ranola et al., 2013).

#### 3.5.1. EGFR pathway restricted by gene expression levels in the NCI60 cell lines

There are sixty unique cell lines of nine tumor types in NCI60 database. We applied the gene expression barcode algorithm (McCall et al., 2011) to the microarray gene expression data of NCI60 cell lines to filter out unexpressed genes. The gene expression barcode is essentially a normalization method leveraging microarray data in the public domain to answer the question: “given an individual microarray experiment of a cell type, is a gene expressed or unexpressed in that cell?” Unexpressed genes are deleted from the default pathway. For each NCI60 cell line, between 40% and 60% of the 235 nodes in the default network made from EGFR pathway were removed after this simple analysis. We then evaluated the importance of nodes or edges in each individual topology.

**Figure 1:**
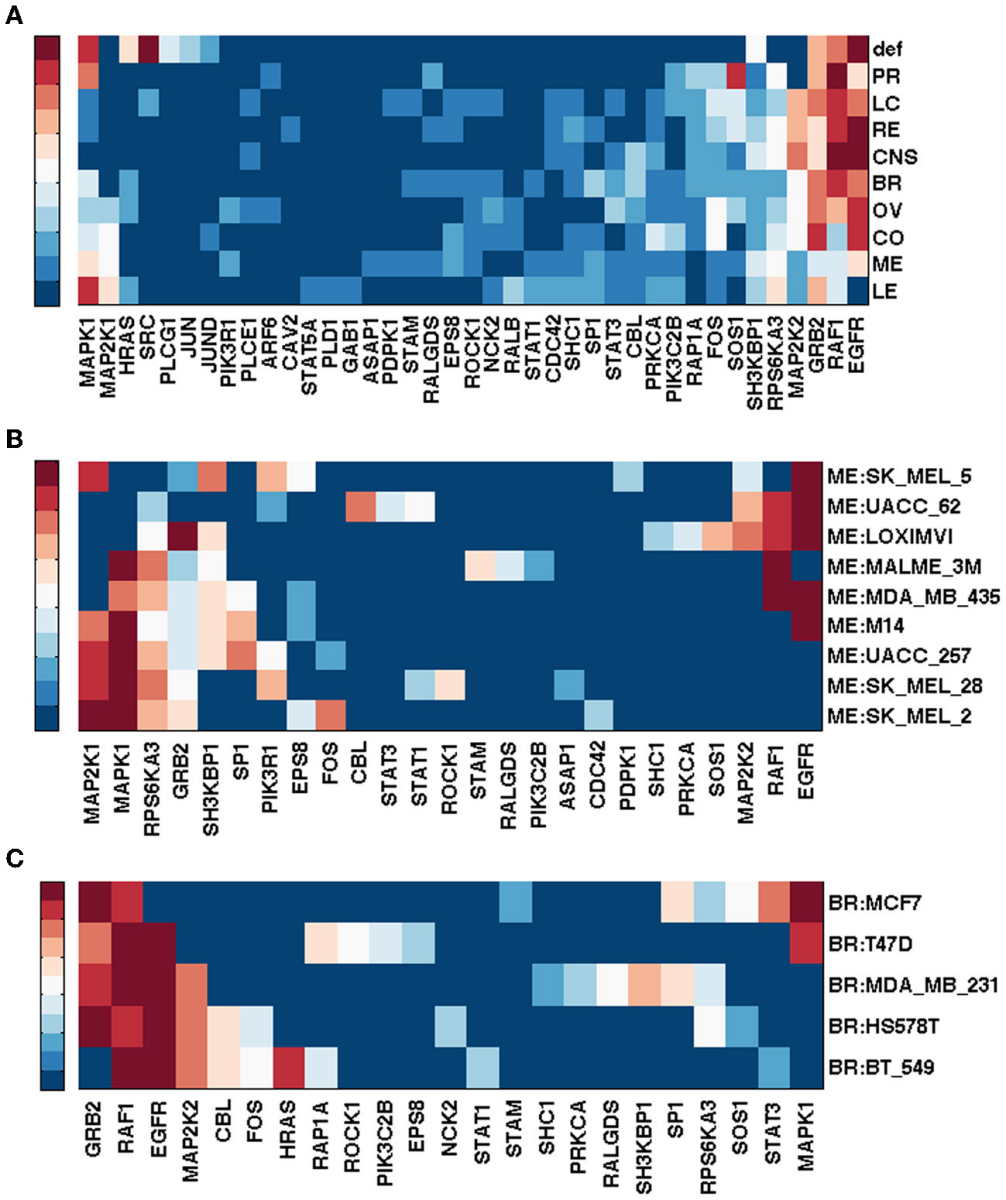
**Top ranked genes by spectral gap centrality with node removal *c*^*r*^_*s*_(*h* = 2) of EGFR pathway conditioned on NCI60 cell line gene expression.** Ranks range from 1 (dark red) to 10 (blue), and > 10 (the darkest blue). **(A)** Rankings are averaged for each tumor type: BR, breast; CNS, central nervous system; CO, colon; LC, non-small cell lung; LE, leukemia; ME, melanoma; OV, ovarian; PR, prostate; RE, renal. def, default pathway with all nodes. **(B)** Gene ranking by *c*^*r*^_*s*_(*h* = 2) for NCI60 melanoma cell lines. **(C)** Gene ranking *c*^*r*^_*s*_(*h* = 2) for NCI60 breast cancer cell lines.

**Figure 2:**
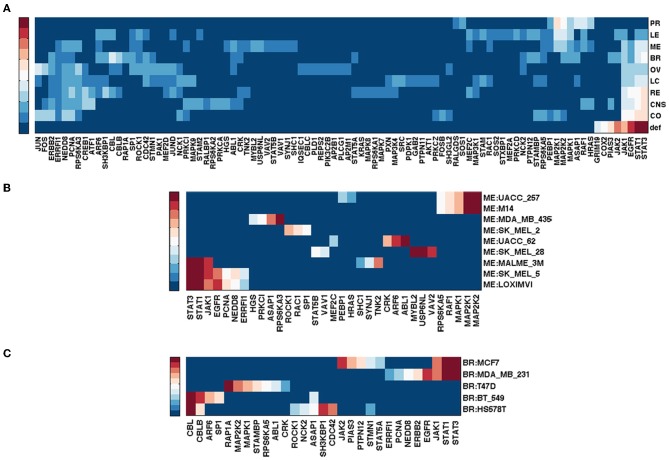
**Top ranked genes by eigenvector centrality *c*_*e*_ of EGFR pathway conditioned on NCI60 cell line gene expression.** Ranks range from 1 (dark red) to 10 (blue), and > 10 (the darkest blue). **(A)** Gene ranking by *c*_*e*_ for tumor types. **(B)** Gene ranking *c*_*e*_ for NCI60 melanoma cell lines. **(C)** Gene ranking *c*_*e*_ for NCI60 breast cancer cell lines.

**Figure 3:**
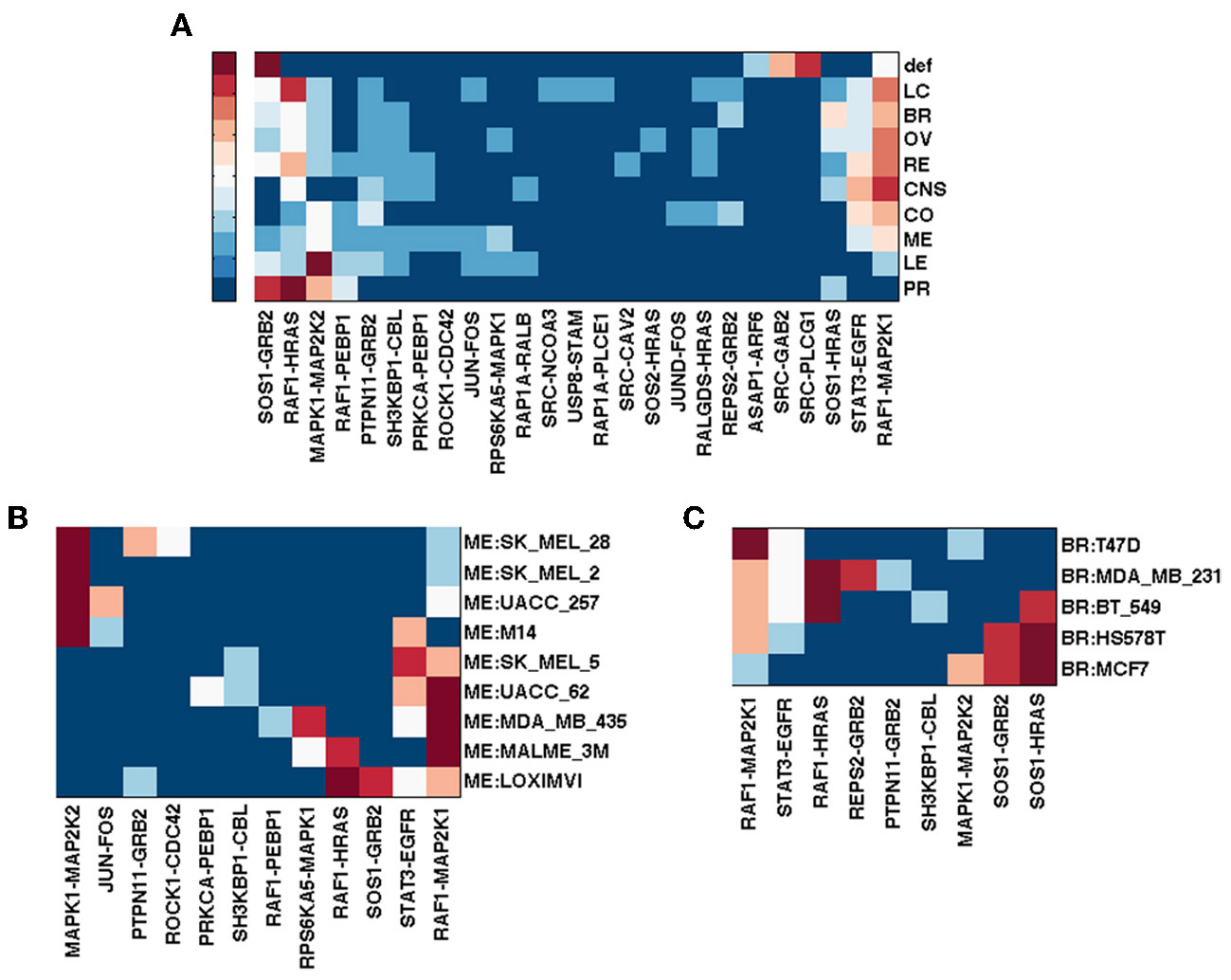
**Top ranked edges by betweenness with edge removal *e*^*r*^_*b*_ of EGFR pathway conditioned on NCI60 cell line gene expression.** Ranks range from 1 (dark red) to 5 (blue), and > 5 (the darkest blue). **(A)** Edge ranking by *e*^*r*^_*b*_ for tumor types. **(B)** Edge ranking by *e*^*r*^_*b*_ for NCI60 melanoma cell lines. **(C)** Edge ranking by *e*^*r*^_*b*_ for NCI60 breast cancer cell lines.

Figure 1A shows gene rankings for the spectral gap centrality measure *c*^*r*^_*s*_(*h* = 2) averaged over cell lines for each tumor type, where the top row labeled as *def* provides the rankings in the default pathway. While all tumor types are different from each other, the patterns of genes expressed and unexpressed in them suggest that a network derived from these genes would be very different from the default pathway. Essentially, each individual cell line presents unique gene expression patterns as well. This diversity requires pathway analyses specific for each individual tumor. Figures 1B,C show the gene rankings for individual melanoma and breast cancer cell lines respectively. Figures 2, 3 show top-ranked nodes by eigenvector centrality and top-ranked edges by betweenness for each tumor type as well as the individual melanoma and breast cancer cell lines.

Melanoma cell lines can be clustered into three groups by *c*^*r*^_*s*_(*h* = 2) top-ranked genes, one with EGFR and RAF1 ranked high exclusively, the other with MAPK1 at top rank, and the third with a mixture of EGFR/RAF1/MAPK1 (Figure 1B). Eigenvector centrality *c*_*e*_ clusters melanoma cell lines quite differently from *c*^*r*^_*s*_(*h* = 2) (Figure 2B). Among breast cancer cell lines, MCF7 is unique when the measure *c*^*r*^_*s*_(*h* = 2) is used to assess the EGFR pathway with GRB2 and MAPK1 ranking highest, while all the other cell lines have EGFR and/or RAF1 as top ranking genes (Figure 1C). T47D appears unique by both *c*_*e*_ and *e*_*b*_ (Figures 2C, 3C). Eigenvector centrality *c*_*e*_ yields unique sets of genes for each cell line except for genes STAT1/3 and CBL, each shared by two cell lines as the top candidates (Figure 2C). CBL protein family has been implicated in a number of human cancers and indeed shown to enhance breast tumor formation by inhibiting tumor suppressive activity of TGF-β signaling (Kang et al., 2012). The application of the edge betweenness measure again clusters melanoma and breast cancer cell lines into two to three groups, but in different ways than those derived with other metrics (Figures 3B,C).

#### 3.5.2. Analysis of the EGFR pathway restricted to eleven normal tissues

**Figure 4:**
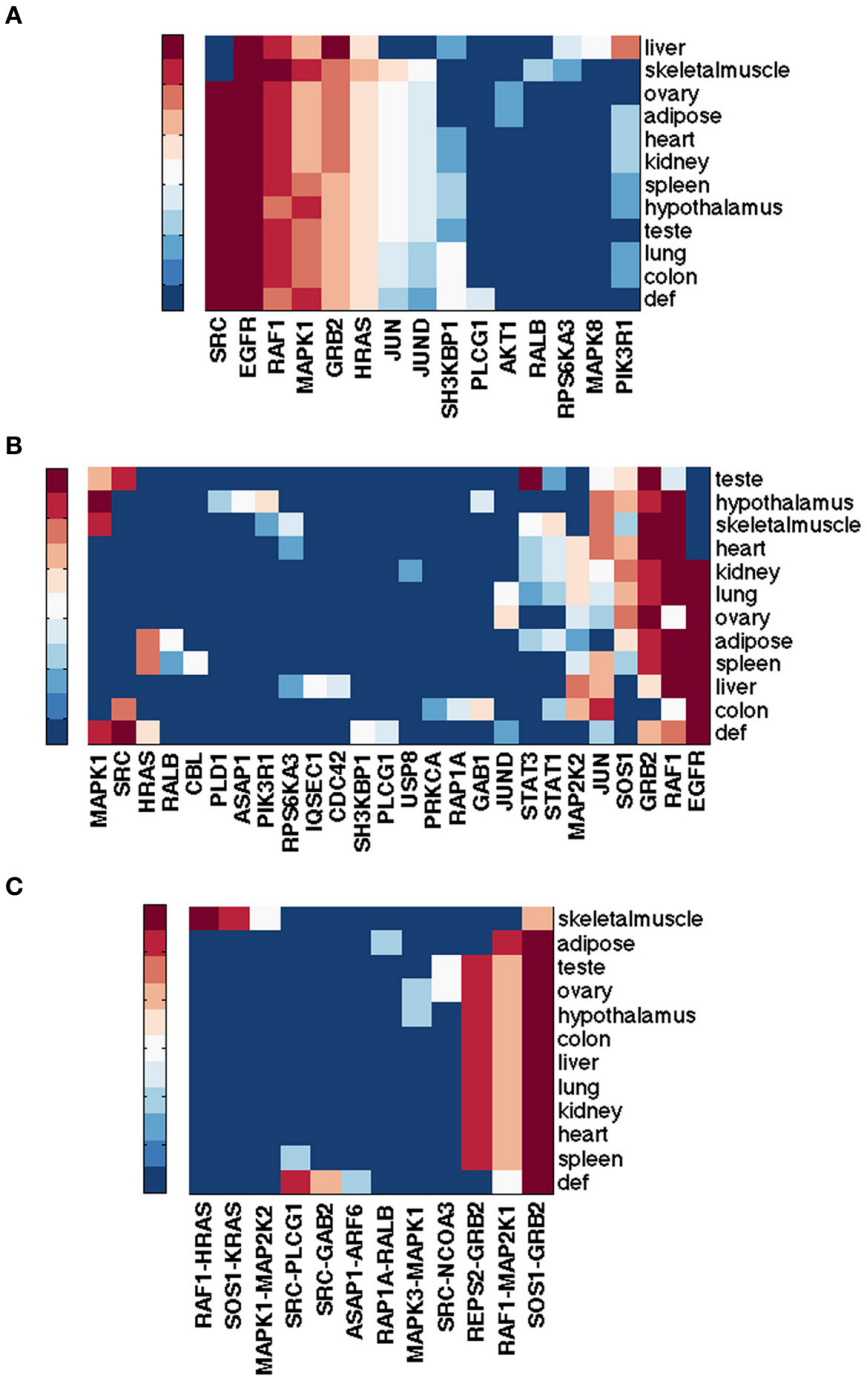
**Top ranked nodes of EGFR pathway conditioned on eleven normal human tissues from RNA-Seq Atlas.** Ranks range from 1 (dark red) to 10 (blue), and > 10 (the darkest blue) for **(A,B)**; from 1 (dark red) to 5 (blue), and > 5 (the darkest blue) for **(C)**. **(A)** Node ranking by spectral gap centrality *c*^*r*^_*s*_(*h* = 2) with RPKM ≥ 0.5. **(B)** Node ranking by spectral gap centrality *c*^*r*^_*s*_(*h* = 2) with RPKM ≥ 3.0. **(C)** Edge ranking by betweenness *e*^*r*^_*b*_ with RPKM ≥ 0.5

The RNA-Seq Atlas (Krupp et al., 2012) has RNA-Seq data for eleven normal human tissues. Hebenstreit et al. (2011) suggests that there are two major classes of gene expression levels in most cells: lowly expressed, which are likely non-functional, and highly expressed, which are likely to be biologically meaningful. The distribution of *log*_2_(RPKM) gene expression values across the eleven human tissues is indeed bimodal, suggesting these two major classes. Determining a simple threshold for defining unexpressed genes, however, is still somewhat arbitrary. We considered a 0.5 RPKM value as a threshold for differentiating unexpressed vs. expressed gene, which is not only often suggested as a conservative threshold, but also seems reasonable in this dataset. Figures 4A,C show the top ranked genes by spectral gap centrality *c*^*r*^_*s*_(*h* = 2) and top edges by the betweenness measure *e*^*r*^_*b*_ for each tissue. With the exception of liver, and to a lesser extent skeletal muscle, the rankings of the most critical genes in the other nine tissues are quite similar to those from the default pathway, and even more similar to each other. The critical edges in tissues differ from those from the default pathway, but they are very similar to each other with the exception of those of skeletal muscle. Even though the data set cannot be compared directly to the NCI tumor cell lines for purely technical reasons, the general patterns of node importance are markedly different (see Figures 1, 3). With the use of a threshold of 0.5 RPKM, around a quarter nodes are deleted from the default EGFR pathway. To make the number of nodes more comparable to the tumor cell lines, we set a more aggressive threshold of 3 RPMK so that between 40% and 60% nodes are filtered out. Figure 4B shows the result for *c*^*r*^_*s*_(*h* = 2) (edge betweenness *e*^*r*^_*b*_ is omitted due to space limitation). Even though there are considerable differences and variations, they are still less varied than the tumor cell types (Figures 1A, 2A, 3A). We note that expression patterns of a tissue could be the averaged expressions over different cell types within the tissue.

#### 3.5.3. Integrated breast cancer pathway restricted by NCI60 breast tumor cells

**Figure 5:**
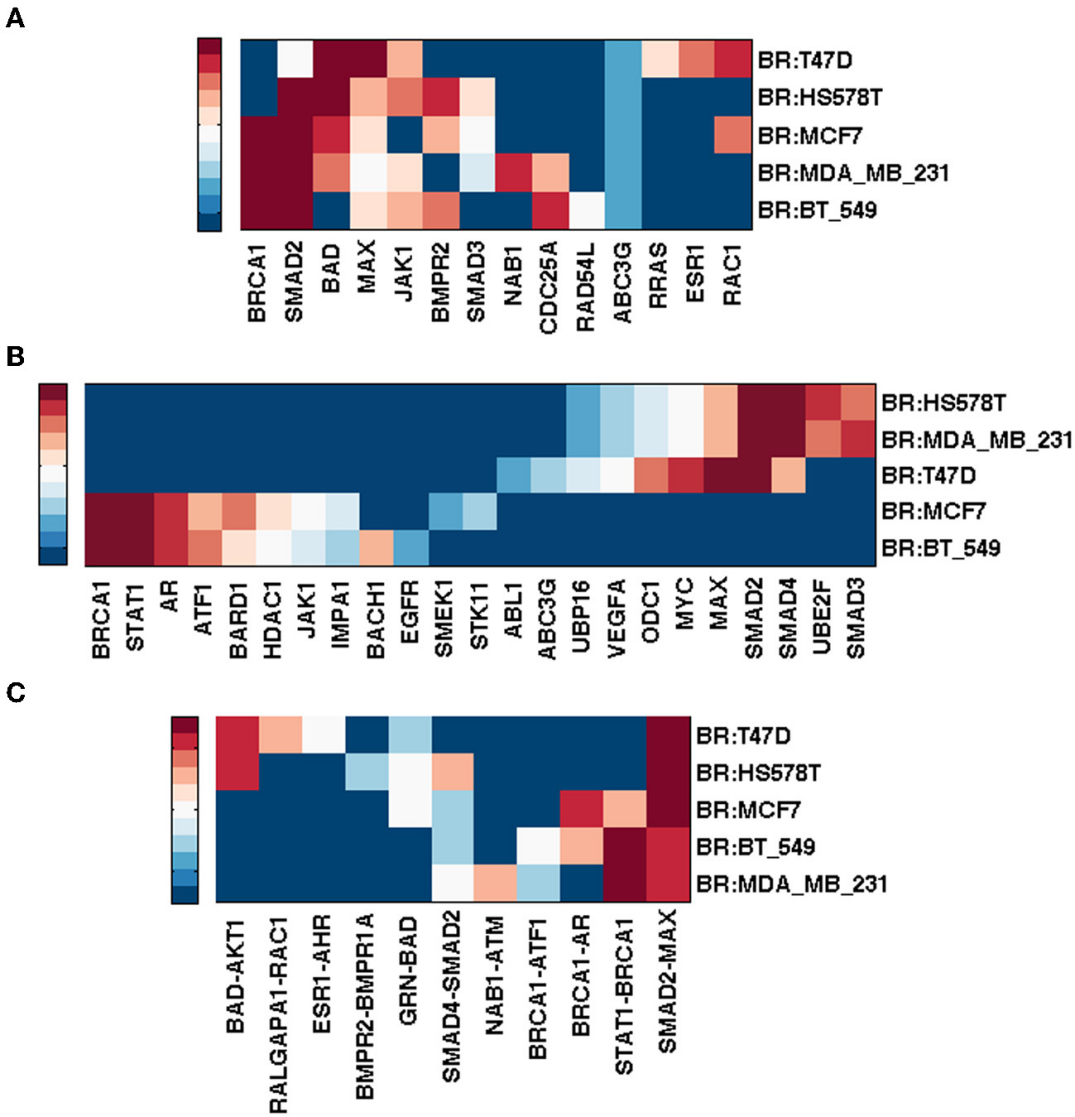
**Top ranked nodes and edges of integrated breast cancer pathway conditioned on NCI60 breast cancer cell lines.** Ranks range from 1 (dark red) to 10 (blue), and > 10 (the darkest blue) for **(A,B)**; from 1 (dark red) to 5 (blue), and > 5 (the darkest blue) for **(C)**. **(A)** Node ranking by spectral gap centrality *c*^*r*^_*s*_(*h* = 2). **(B)** Node ranking by eigenvector centrality *c*_*e*_. **(C)** Edge ranking by betweenness *e*^*r*^_*b*_.

The Integrated Breast Cancer pathway incorporates the most important proteins for breast cancer. It has 190 unique nodes and 348 edges (mean node degree = 1.83; graph density = 0.02). Figure 5 shows the top ranking nodes and edges by different measures for each NCI60 breast cancer cell line.

While BRCA1 ranked highest by *c*_*d*_ (not shown), *c*^*r*^_*s*_(*h* = 2, 3) (*h* = 3 not shown) for cell lines MCF7, MDA_MB_231 and BT_549, MAX (Myc associated factor X) ranked highest by *c*_*d*_ and *c*^*r*^_*s*_(*h* = 3) for HS578T and T47D. It is known that MYC deregulation contributes to breast cancer development and progression. Loss of BRCA1 coupled with MYC overexpression leads to the development of breast cancer (Xu et al., 2010) and recent evidence has shown that MYC is druggable (Pourdehnad et al., 2013).

Smad2 ranked high by at least one measure for each cell line. Smad genes are highly ranked by *c*_*e*_ for all but MCF7 and BT_549, for which STAT1 and AR emerged more important (in addition to BRCA1). Although Smad2/3/4 signaling plays a tumor suppressor role, it also exhibits a pro-metastatic function in breast cancer (Kang et al., 2005). It is also believed that Smad-dependent pathway is involved in TGF-β tumor suppressor functions. Various TGF-β inhibitors are in development and preclinical studies have shown their promises in cancer treatments (Nagaraj and Datta, 2010).

Evidence has correlated up-regulation of STAT1 activity with increased breast tumor progression and immune suppression in tumor microenvironment, thus STAT1 inhibition is a promising immune therapeutic target (Hix et al., 2013). Androgen receptor (AR) is commonly expressed in breast cancers. It ranked high by *c*_*e*_ for cell lines MCF7 and BT-549. There is a history of targeting AR for therapy in breast cancer, although the efficacy of AR targeted treatments is moderate (Garay and Park, 2012) probably due to a lack of clear understanding of the AR signaling mechanism. For MCF7 cell line though, inhibitory effects of androgens targeting AR have indeed been shown in multiple studies (Greeve et al., 2004; Macedo et al., 2006).

Notice that since the analysis of the breast cancer pathway is conditioned on the gene expression patterns in each cell line, major tumor suppressor genes such as P53 and BRCA2 are deleted. The exome data of NCI60 (Abaan et al., 2013) cell lines shows that each of the five breast cancer cell lines has between one to four missense or silencing TP53 mutations, and two to five missense or silencing mutations in BRCA2. Only MDA_MB_231 has a silencing BRCA1 mutation. If we analyze the default breast cancer pathway instead of the pathways built only from genes expressed in the cell lines, the top three gene nodes are P53, AKT1 and BRCA1 based on the *c*_*d*_ or *c*_*e*_ measures, or CERK1, SMAD2 and AKT1 by the *c*^*r*^_*s*_(*h* = 2) measure, respectively. The top two ranked edges based on the betweenness measure (with edge removal) are the TGFR1-SMAD4 and P53-C9JNK1 edges.

## 4. Discussion

The identification of genes that are optimal or logical therapeutic targets in tumors based on genomic information is crucial for *individualizing* cancer treatments. We explored the utility of network centrality analysis of standard pathways and pathways based on gene expression information in identifying potential therapeutic targets for a tumor. We also described the complexity of, and issues associated with, such analysis. We considered ranking genes in a network or pathway either by their centrality values or by iteratively recording the top-ranked node and reevaluating the remaining subnetwork with the highest ranked node removed. When analyses are performed on PPI subnetwork created from genes associated with a specific pathway, the top ranked genes based on different node importance measures are highly positively correlated. We observed a similar phenomenon when PPI subnetworks derived from genes that have been implicated in particular types of cancers were assessed, both when using the genes in these PPI subnetworks alone and by expanding these subnetworks by including nodes one or two edges from the seed genes used to create the PPI subnetwork (data not shown). The high-degree nodes in a PPI network are critical to the functioning of that network, and thus are likely to be important drug targets. However, such nodes are not likely to be specific to a particular pathway and as such targeting them therapeutically could also be potentially toxic to a patient.

When applied to a signaling pathway, various measures of centrality yield different sets of important genes and the rankings of these genes across different node importance measures are much less correlated. This lack of correlation among node importance measures may provide more insight into the functioning of a network or pathway since the different measures may be capturing different aspects of information flow through the network. However, a possible confounding factor in the analysis of node importance in networks is that the same pathway may be represented in different ways across different databases, leading to different network topologies. It is unclear how to determine which topology is the best representation of a pathway in such cases.

In the context of different measures of node importance, eigenvector centrality has the potential to reveal nodes that may impact other highly influential nodes (for instance nodes of high degree). These other nodes may reflect genes that could serve as alternative therapeutic targets when the highest ranked nodes or genes are hard to target or possibly be toxic to the system as a whole if targeted therapeutically directly. Identifying these alternative important nodes using eigenvector centrality should be done on the pathway without iteratively deleting nodes or those alternative nodes are not likely to be discovered. For instance, while SRC, STAT3, EGFR and GRB2 ranked the highest in the EGFR pathway by two or more measures, STAT1, JAK1/2, PIAS3, COX2 and GRIM19, all being neighbors of STAT3, ranked within top ten exclusively by the eigenvector centrality. Each of these genes has been implicated in some type of cancers and some are known targets of cancer treatments. As mentioned in Section 3.2, Ruxolitinib, an FDA-approved drug for treatment of a type of bone marrow cancer, is a JAK1/2 inhibitor (Mesa, 2010). NSAIDs and Celecoxib are COX2 inhibitors and have protective effects against colorectal and breast cancers (Gupta and DuBois, 2001; Arun and Goss, 2004; Brown and DuBois, 2005). In addition, Hide et al. (2011) showed that the combination of a PTGS2 (COX2) inhibitor and an EGFR inhibitor prevented tumorgenesis of oligodendrocyte lineage-derived glioma-initiating cells. Finally, Li et al. (2013) demonstrated that microRNA-26b might act as a tumor suppressor in breast cancer by targeting PTGS2.

Nodes ranked high by the betweenness measure with iterative node removal are often on parallel cascades in the pathway, which are important for simultaneously targeting multiple pathways in cancer treatment. The top three nodes identified in this fashion in MAPK pathways, for example, are MEK2, JNK and ASK1, which reside on ERK1/2, JNK and p38 cascades respectively. Edge betweenness generates potential edge-specific, or edgetic targets, which are more specific to a particular pathway and the nodes implicated in these edges might provide an alternative for therapeutic targeting if the highest ranked individual nodes are hard to target. Similarly, edges identified as important by iterative edge removal tend to reside on separate paths.

Although high degree nodes are very important to the functioning of a network, they are also more prone to differ if local changes in a network topology are made. The spectral gap centrality measure, on the other hand, is less sensitive to local degree changes, and is more reliable if slightly different network topologies are considered. The spectral gap centrality measure also captures both degree and betweenness phenomena simultaneously, thus complementing betweenness measures when used in isolation in an important way. This is particularly true in the context of signaling pathways where the betweenness measures tend to capture fragile nodes and edges. The choice of the parameter *h* in the spectral gap centrality measure calculation is more complicated and is likely best approached empirically. Smaller values of *h* tend to capture local node importance while larger values of *h* tend to capture more global node importance. For typical pathways and PPI networks, setting *h* = 2 or 3 is a reasonable choice. The spectral gap centrality measure node rankings are also more informative when computed with iterative node removal.

Ultimately, in the context of finding potential therapeutic targets for tumors, we firmly believe that network analysis should consider cell or tissue specific pathways and networks and not rely on generalized or tissue independent canonical pathways and networks. In order to assess tissue-specific networks and pathways, we considered the use of the expression levels of genes in tissues to filter out unexpressed genes. We did this by using either gene expression barcodes based on available array data or a RPKM threshold based on RNA-Seq data. When different measures of node importance are applied to tissue or cell-specific pathways obtained in this way, the resulting top-ranked genes varied significantly among different cell types. We found that variations in node importance between different tumor types are generally larger than those variations between different normal tissues. This is to be expected given the complex rearrangements and perturbations in tumors. For a particular tumor type, analysis of different tumor cell lines or subtypes results in different nodes deemed crucial or important to a particular pathway. For instance, when the integrated breast cancer pathway is restricted by the five NCI60 breast tumor cell lines based on their respective gene expressions, BRCA1 ranked highest by degree and spectral gap centralities for cell lines MCF7, MDA_MB_231 and BT_549, while MAX ranked highest by the same measures for cell lines HS578T and T47D. SMAD2 ranked high by at least one centrality measure for each of the five cell lines. While SMAD genes were highly ranked by eigenvector centrality for MDA_MB_231, HS578T and T47D, STAT1 and AR appeared more important for MCF7 and BT_549.

We recognize that there are limitations and caveats in our analyses. As more and more RNA sequencing studies are being pursued on tumors, a simple threshold used to differentiate expressed and unexpressed genes in these tumors will be harder to define. Thus, better methods need be explored to determine which genes might need to be filtered out or included in a pathway analysis. While capturing important relevant oncogenes or genes impacting oncogenes in a pathway, filtering genes based on whether they are expressed or unexpressed in a cell type naturally filters out abnormally silenced genes, thus potentially excluding malfunctioned tumor suppressor genes in analysis, such as the p53 gene. This can be salvaged by analyzing the default pathway to some extent. In this light, given the extremely complex nature of cancers, finding critical genes in specific pathways is just a tiny piece of a puzzle to determine how best to treat cancers. Not only will an analysis of critical nodes in a network need be approached with caution, but it should also be used in conjunction with other information, such as the analysis of DNA sequence mutations, copy number variations and other bio-markers. In addition, treating gene expression as a binary factor to construct a network's topology for use in an analysis of node importance is admittedly a simplistic approach. Rather, expression levels and rates of gene amplifications can also be incorporated into network analysis. Also, in addition to analyzing tumor cells alone, it will likely be more informative to compare normal and tumor samples to better quantify tumor-specific genomic perturbations. Ultimately, we believe our analyses shed light on the utility of measures of node and edge importance in an analysis of gene networks and pathways in tumor biology and cancer treatment choice and hope that they may motivate further research in this area.

### Conflict of interest statement

The authors declare that the research was conducted in the absence of any commercial or financial relationships that could be construed as a potential conflict of interest.
